# A Possible Connection Between Plant Longevity and the Absence of Protein Fibrillation: Basis for Identifying Aggregation Inhibitors in Plants

**DOI:** 10.3389/fpls.2019.00148

**Published:** 2019-02-13

**Authors:** Hossein Mohammad-Beigi, Lars Kjaer, Hoda Eskandari, Farhang Aliakbari, Gunna Christiansen, Gianluca Ruvo, Jane L. Ward, Daniel Erik Otzen

**Affiliations:** ^1^Interdisciplinary Nanoscience Centre (iNANO), Aarhus University, Aarhus, Denmark; ^2^Department of Industrial and Environmental Biotechnology, National Institute of Genetic Engineering and Biotechnology, Tehran, Iran; ^3^Department of Biomedicine-Medical Microbiology and Immunology, Aarhus University, Aarhus, Denmark; ^4^Computational and Analytical Sciences Department, Rothamsted Research, Harpenden, United Kingdom; ^5^Department of Molecular Biology and Genetics, Aarhus University, Aarhus, Denmark

**Keywords:** plant extracts, plant longevity, protein aggregation, inhibitors of aggregation, aggregate toxicity, α-synuclein, beta amyloid

## Abstract

The ability of proteins to aggregate to form well-organized β-sheet rich amyloid fibrils is increasingly viewed as a general if regrettable property of the polypeptide chain. Aggregation leads to diseases such as amyloidosis and neurodegeneration in humans and various mammalian species but is also found in a functional variety in both animals and microbes. However, there are to our knowledge no reports of amyloid formation in plants. Plants are also the source of a large number of aggregation-inhibiting compounds. We reasoned that the two phenomena could be connected and that one of (many) preconditions for plant longevity is the ability to suppress unwanted protein aggregation. In support of this, we show that while protein extracts from the sugar maple tree *Acer saccharum* fibrillate readily on their own, this process is efficiently abolished by addition of small molecule extracts from the same plant. Further analysis of 44 plants showed a correlation between plant longevity and ability to inhibit protein aggregation. Extracts from the best performing plant, the sugar maple, were subjected to chromatographic fractionation, leading to the identification of a large number of compounds, many of which were shown to inhibit aggregation *in vitro*. One cautious interpretation is that it may have been advantageous for plants to maintain an efficient collection of aggregation-inhibiting metabolites as long as they do not impair metabolite function. From a practical perspective, our results indicate that long-lived plants may be particularly appropriate sources of new anti-aggregation compounds with therapeutic potential.

## Introduction

Since the first discovery of plaque in the brains of patients suffering from Alzheimer’s disease (Masters et al., 1985), a number of proteins have been shown to form insoluble β-sheet-rich structures called amyloid or fibrils under physiological conditions (Chiti and Dobson, 2006). Among these are proteins related to common diseases of lifestyle and aging such as type II diabetes (Pillay and Govender, 2013), Alzheimer’s disease, and Parkinson’s disease (Poggiolini et al., 2013) as well as rare genetic disease such as FAS4 mediated corneal dystrophy (Han et al., 2010; Stenvang et al., 2018). In addition, a growing number of microbial organisms have been shown to form functional amyloid (Otzen and Nielsen, 2008; Dueholm et al., 2013). Amyloid formed by proteins such as CsgA (*Escherichia coli*) (Chapman et al., 2002) and FapC (*Pseudomonas* sp.) (Dueholm et al., 2010) strengthens biofilms and promotes cell–cell communication, while fungal hydrophobins can form two-dimensional amphipathic amyloid films at air–water interfaces that allow growing fungi to overcome interfacial tension as well as providing immune-evasive cover (Bayry et al., 2012). The widespread occurrence of fibrillation has prompted the hypothesis that fibrillation of proteins is a normal (“generic”) property (Dobson, 1999) and in fact may represent a more stable form than the monomeric folded state (Baldwin et al., 2011). Nevertheless, to date there are very few cases of protein fibril formation in plants, and with few exceptions such as the rubber latex protein Hevb1 (Berthelot et al., 2012), the few reports available do not represent authentic fibrillation of endogenous proteins *in planta* (Antonets and Nizhnikov, 2017). Examples limit themselves to a tobacco plant expressing a foreign protein (from maize) in the chloroplast (Villar-Pique et al., 2010), a coconut antimicrobial peptide which can fibrillate in aqueous buffer (Gour et al., 2016), a soya β-conglycinin subunit which fibrillates *in vitro* after heat treatment (Wang et al., 2011), and a protein fragment that fibrillates *in vitro* (Garvey et al., 2013).

In contrast, there is an extensive literature on the fibrillation-inhibiting effects of plant extracts, including both those consumed as part of a healthy diet (Modi et al., 2015; Casamenti and Stefani, 2017) and others found in traditional Eastern (Rajan et al., 2015; Liu et al., 2016; Malishev et al., 2017) and Western (Tunón, 1995; Lobbens et al., 2016) medicines. A recent review (Velander et al., 2017) concluded that more than half of the 72 known natural product inhibitors of aggregation were polyphenols (e.g., flavonoids, anthraquinones, alkaloids, and terpenes), and included compounds such as oleuropein and oleocanthal from olive oil, resveratrol (from fruit and red wine), curcumin (from turmeric), as well as EGCG and myricetin (green tea). These molecular groups are also among the best performers in broader surveys spanning different chemical classes (Masuda et al., 2006). Interestingly, the vast majority of the inhibitors are found in perennial spices (Ono et al., 2004; Ishigaki et al., 2013) and tea (Palhano et al., 2013). The shorter-lived annual crops do not appear to harbor the same inhibitory components. A study comparing ethyl acetate extracts from vegetables and spices confirmed that spices are much more effective than the short-living vegetables (Fuentes et al., 2016). The very absence of such data in general might indicate that these otherwise ubiquitous plants have few or no such inhibitors.

The differences observed between extracts of annual and perennial plants prompted our investigation of the relationship between plant longevity and fibrillation inhibition. We reasoned that the absence of fibrillation in plants is caused by the presence of small-molecule fibrillation inhibitors, particularly in long-lived plants. (We emphasize that we do not claim that the primary evolutionary driving force for the development of these small molecules in plants is their anti-aggregation properties, but that such properties could constitute collateral benefits in terms of, e.g., longevity and could from a pragmatic view be useful sources of new fibrillation inhibitors.) As a corollary, in the absence of fibrillation inhibitors, plant proteins should be able to fibrillate *in vitro* under the same conditions as many mammalian proteins. Here, we examine this hypothesis. We start by showing that plant proteins can indeed fibrillate on their own but are inhibited by small molecule extracts from the same plant. We then analyze extracts from a large number of different plants to examine the correlation between longevity and the ability to inhibit fibrillation of model proteins. Finally, we identify a number of inhibitors in the extract of the most promising plant candidate, several of which have already been documented to act against fibrillation. We find that there is a certain tendency for metabolites from longer lived plants to be particularly efficient at inhibiting protein aggregation, although there is unlikely to be a simple causal relationship since some short-lived plants also show efficient aggregation inhibition. Nevertheless, whatever the evolutionary underpinnings, our anti-aggregation tests of plant extracts as a simple but effective source of new potential aggregation inhibitors.

## Materials and Methods

### Materials

All chemicals were of the highest purity possible and were from Sigma–Aldrich unless otherwise stated. Deuterated NMR solvents were from Goss Scientific (Crewe, United Kingdom). Aβ peptide and amylin peptide were purchased from Chinese Peptide at 95% purity and verified by in-house MALDI-TOF. αSN was prepared recombinantly from *E. coli* as described (Lorenzen et al., 2014a). All plant material was obtained from the Aarhus Botanical Gardens (Table 1). We saw similar inhibitory activity for *A. saccharum* leaves harvested in the spring and the fall. We avoided harvesting mature spring leaves (i.e., not light green) and senescent fall leaves.

**Table 1:** Dosage of plant extract required to completely inhibit the fibrillation of 1 mg/mL αSN^a^.

Plant	Dosage per 150 μl well (μl)	Minimum effective mass (*m*_me_) per 150 μl well (μg)^b^	*m*_me_ relative to total inhibition with EGCG^c^
*Acer saccharum*	1	9.4	0.20
*Acer saccharinum*	1	17	0.36
*Pseudosuga menizies*	10	169	3.6
*Eucalyptus globolus*	10	67	1.4

*^*a*^Complete inhibition defined as having a ThT level that does not significantly exceed that of the ThT fluorescence level seen in the lag time of the control experiment (αSN without any extract). ^*b*^Based on vacuum drying and weighing of 500 μl extract (contribution from buffer subtracted). ^*c*^Weight required for total inhibition relative to that required for total inhibition with EGCG*.

### Small Molecule Extraction

Around 2 mL of plant material was ground in liquid nitrogen and transferred to one or more 2 mL Eppendorf tubes, which were then filled with acetone (or other solvent as detailed below) and heated up to 60°C for 15 min, followed by overnight incubation at 4°C. The samples were centrifuged for 10 min at 16,000 *g* and the supernatant collected. The pellet was again washed with acetone or other solvent, incubated for 15 min and centrifuged for 10 min at 16,000 *g*, and the supernatant was added to the previous extract. The supernatant was evaporated under vacuum overnight and redissolved in 2 mL of 100 mM Tris buffer pH 8.5. The sample was filtered through a 0.2 μL filter, followed by a 3 kDa amicon spin filter. All extracts were stored at -20°C. For initial fibrillation inhibition assays used to screen the 49 different organisms, the extract was suspended in a volume equivalent to the original volume of plant tissue, and 10 μL hereof were added to a final volume of 150 μL in the plate. This 15-fold dilution was used in all plant extracts, unless otherwise specified. For dose–response studies with four selected plants (*A. saccharum, Acer saccharinum, Pseudotsuga menziesii*, and *Eucalyptus globolus*) volumes between 0.25 and 10 μl were used. Regarding choice of solvent for extraction, we note that we attempted extraction both with ethanol, water, acetone, 50% acetone/water (v/v), and 80% ethanol/water (v/v). Acetone extract was much more potent than the other extracts in terms of inhibitory properties; further, HPLC analysis of the different extracts revealed the same peaks across the different extraction methods, but with the acetone extract containing far higher concentrations. Finally, acetone was the most volatile solvent, making it the easiest to evaporate.

### Fractionation of *Acer saccharum* Extract

The small molecule extract was fractionated on a reverse phase Jupiter C18 (250 × 10 mm) column using a gradient of 5–35% acetonitrile over the first 45 ml, followed by a 35–90% gradient of acetonitrile over 5 ml and 90% acetonitrile over the next 5 ml. The other mobile phase was demineralized water; 0.1% trifluoroacetic acid was present in both phases and a flow rate of 1 ml/min was used.

### Purification of Plant Protein

*Acer saccharum* (Sugar maple) leaves were ground under liquid nitrogen with a mortar and pestle until a 50 mL tube was filled with ground material (uncompressed). The tube was then filled with a solution containing 1 mM EDTA, 4 mM DTT, 2 mM PMSF, 0.1% Tween 20 and 2% polyvinylpyrrolidone (PVP), and 100 mM Tris buffer pH 8.5 and incubated on a shaker at 37°C for 1 h. The material was centrifuged at 9,000 *g* for 20 min to pellet plant material after which the supernatant was filtered through a coffee filter and ultracentrifuged at 150,000 *g* for 1 h. The supernatant was filtered sequentially through filters of pore size 2, 0.45, and 0.1 μm using a vacuum filtration system. The buffer was replaced with 100 mM Tris pH 8.5 through three rounds of concentration to 1/10 volume with an Amicon stirred cell filtration system with a 3 kDa filter, followed by dilution to the original volume. In the final round, the resultant product was volume-reduced to 5 mL and fractionated on a 24 mL Superose 6 column; 0.25 ml fractions were collected and concentrated fivefold using 3 kDa cut-off Millipore spin filters.

### Extraction of Insoluble Plant Protein Aggregates

In order to extract the insoluble proteins from *Acer* leaves, 2 g *A. saccharum* leaves were thoroughly pulverized in liquid nitrogen and then incubated with 50 ml of extraction buffer (20 mM Tris-HCl pH 7.5, 150 mM NaCl, 0.1 mM PMSF) at 37°C for 15 min. To remove cell debris, the homogenate was centrifuged at 100 *g* for 15 min and filtered through a coffee filter. RNAase and DNAase 1 mg/ml were added and the mixture was incubated at 37°C for 30 min. Protein aggregates were separated by centrifugation at 8,000 *g* for 15 min at 37°C and the supernatant was discarded. The aggregates were washed once with the same buffer containing 0.5% Triton X-100 and twice with sterile PBS. The pellet was saved and kept at -20°C until analysis. The frozen pellets were resuspended in extraction buffer.

### Fibrillation of Proteins

Plant proteins were diluted to 1 mg/ml [using an average extinction coefficient at 280 nm of 1.0/(mg ml^-1^ cm^-1^)] in 100 mM Tris pH 8.5 and incubated with 40 μM ThT at 37°C (based on a stock of 5 mM ThT in ethanol). Plate-reader-based fibrillation of individual proteins was carried out a concentration of 1 mg/ml αSN, 40 μM Aβ peptide (from a 6 mM stock in DMSO based on A_280_), and 10 μM amylin (dissolved in 100 mM Tris pH 8.5 at 100 μM). For all four protein groups, each well was filled with 150 μl solution including 40 μM of ThT, 100 mM Tris-Cl pH 8.5, and different amounts of plant extract or individual plant inhibitors. Plant proteins and αSN were fibrillated in a Genios Pro (Tecan, Männedorf, Switzerland) plate reader (five reads per data point with 40 μs integration time, reads every 12 min including 10 min orbital shaking at 37°C with one glass bead), while Aβ and amylin (which did not require shaking to induce fibrillation) were fibrillated in a Varioscan flash plate reader (reading every 10 min, bottom reads, 12 nm bandwidth, measurements time 100 ms without shaking or glass beads). For both readers, excitation was at 448 nm and emission at 485 nm. After fibrillation, the solution was centrifuged at 50,000 *g* for 1 h and the pellet used for EM visualization.

### Procedure for Incubation With Seeds

Two to four milligrams/milliliter αSN was allowed to fibrillate to completion by incubation at 37°C with 200 rpm shaking for 48 h. Afterward, the fibrils were centrifuged for 30 min at 16,000 *g* on a bench top centrifuge. αSN concentration was measured in the supernatant by absorption at 280 nm (αSN extinction coefficient 5,600 M^-1^ cm^-1^). The amount of fibrils was calculated as the decrease in mass compared to absorption before fibrillation. The fibrils were resuspended in the original volume and sonicated using a Qsonica sonicator with 1/16” tip at 20% intensity with cycles of 5 s on/5 s off for 1 min. These seeds were added at different ratios to 1 mg/ml freshly prepared monomer and incubated on a Varioscan flash plate reader using excitation at 444 nm and emission at 485 nm at 37°C with a reading every 10 min using bottom optics. To test the effect of extracts or small molecules, 1 μl plant extract or 30 or 150 μM of the tested compound was added to the solution containing 1 mg/ml monomeric αSN and 5% seeds.

### Oligomer Preparation

This was carried out on an ÄKTA Basic system. Solutions of 3 mg/mL of αSN were incubated on a shaking incubator for 5 h at 37°C. The solution was then filtered and separated on a 20 cm Superose 6 column with PBS buffer at a flow rate of 0.5 mL/min, while monitoring absorbance at 215 nm. Oligomers were collected in 0.5 mL fractions and concentrated in 15 mL Amicon spin filters with a 10 kDa cut-off (the αSN oligomer has an average mass of ca. 420 kDa; Lorenzen et al., 2014a).

### Calcein Release

Calcein-filled vesicles were prepared by suspending 10 mg/mL of DOPG and 40 mM calcein in milliQ water. The solution was then frozen in liquid nitrogen, thawed in a water bath 10 times, and extruded 20 times through a 200 nm filter. The solution was passed over a PD 10 column (GE Healthcare Europe GmbH, Brondby, Denmark), the progress of the solution through the columns was observed and fractions were collected when the first colored solution came through the column. The first 0.5 mL was discarded and the second 0.5 mL was collected for the assay. We estimate the vesicle concentration in this fraction to be 5 mg/ml.

The final concentration of oligomer in calcein assays was 0.029 mg/ml (2 μM in monomer αSN equivalents); 5 μL of extract, or fractionated extract equivalent in amount to 5 μL of the original extract, was mixed with oligomer solution to 148 μL, incubated for 1 h, after which 2 μL of calcein-filled vesicles was added. Immediately after mixing, fluorescence *F*_0_ was measured on a Varioskan Flash fluorimeter (Thermo Scientific) with excitation at 495 nm and emission at 515 nm, 5 nm bandwidth, and 100 ms measurement using top optics with autorange. After 40 min measurement, fluorescence *F*_40_ was recorded, 1 μL of 1% Triton X-100 was added to each well to ensure complete vesicle lysis, and the fluorescence was allowed to stabilize over 10 min after which *F*_final_ was recorded. The calcein release *R* was calculated as follows:

$$R=(F_{40}-F_{0})/(F_{final}-F_{0})$$

### UHPLC-MS Analysis

Ultrahigh-performance liquid chromatography-mass spectrometry (uHPLC-MS) was recorded with an Ultimate 3000 RS uHPLC system, equipped with a DAD-3000 photodiode array detector, coupled to an LTQ-Orbitrap Elite mass spectrometer (Thermo Scientific, Germany). UHPLC separation was carried out using a reversed-phase C_18_ Hypersil gold column (1.9 μm, 30 mm × 2.1 mm i.d., Thermo, Hemel Hempstead) which was maintained at 35°C. The solvent system consisted of water/0.1% formic acid (A) and acetonitrile/0.1% formic acid (B). Separation was carried out in 30 min under the following conditions: 0 min, 0% B; 27 min, 70% B; 28 min, 100% B. The flow rate was 0.3 mL/min and the injection volume was 10 μL. Mass spectra were collected using an LTQ-Orbitrap Elite with a heated ESI source (Thermo Scientific, Germany). Mass spectra were acquired in negative mode with a resolution of 120,000 over *m/z* 50–1,500. The source voltage, sheath gas, auxiliary gas, sweep gas, and capillary temperature were set to 2.5 kV, 35 (arbitrary units), 10 (arbitrary units), 0.0 (arbitrary units), and 350°C, respectively. Default values were used for other acquisition parameters. Automatic MS-MS was performed on the three most abundant ions and an isolation width of *m/z* 2 was used. Ions were fragmented using high-energy C-trap dissociation (HCD) with a normalized collision energy of 65 and an activation time of 0.1 ms. Data analysis was carried out using Xcalibur v. 2.2 (Thermo Scientific, Germany).

### CD Spectroscopy

To study whether the identified small molecules affect the binding of αSN to vesicles, 14 μM monomeric αSN was incubated with 0.3 mM vesicles of DMPG of size 100 nm (prepared by extrusion as described in Kjær et al., 2009) in the presence of small molecules (40 μM) identified from the plant extract. Temperature scans were recorded as changes in ellipticity at 222 nm with a scanning speed of 60°C/h on a Jasco J-810 spectrophotometer (Jasco Spectroscopic Co., Ltd., Hachioji City, Japan) using a 1-mm cuvette (Kjær et al., 2009).

### Cell Viability Assay

The MTT assay was used to measure the cell viability after 24 h treatment with monomeric or aggregated form of αSN. OLN-93 cells were seeded in 96-well plates at a density of 3 × 10^4^ cell/ml in RPMI 1640 supplemented with 10% FCS, 100 units/ml penicillin, and 100 μg/ml streptomycin. The cells were incubated in a humidified atmosphere incubator with 5% CO_2_ and 95% humidity at 37°C for 24 h. Media was then replaced with fresh media containing 10% αSN samples with or without compounds (collected during the fibrillation process) and incubated for more 24 h. Then the media was replaced with fresh media containing 10% MTT (5 mg/ml) and the cells were incubated for an additional 4 h at 37°C. As a final point, the MTT solution was removed and replaced by 100 μl DMSO in order to dissolve formazan crystals through incubating for 1 h on a shaking table at room temperature. Ultimately, absorbance was determined by using a plate reader at 570 nm.

### ROS Assay

To assess whether αSN aggregates could interfere with the level of ROS within the cells, DCFH-DA was used. OLN-93 cells were seeded in 96-well plates at a density of 6 × 10^4^ cells/ml and incubated in a humidified atmosphere incubator at 37°C for 24 h. The media were then removed and the cells washed with PBS and renewed with PBS containing 15 μM DCFH-DA. The plate was incubated for 45 min in dark in CO_2_ incubator. DCFH-DA was then removed and the wells were washed with PBS. The cells were then treated with culture media containing αSN aggregates and incubated at 37°C. The fluorescence intensities of DCF were then recorded using an automatic microtiter plate reader (emission/excitation: 490/527 nm).

## Results

### Proteins From *Acer saccharum* Form Fibrils Upon Prolonged Incubation Unless Small-Molecule Plant Extract Is Added

Our hypothesis posits that plants do not form fibrils because they contain inhibitors. As a test case, we selected *A. saccharum*, both for reasons of availability and convenience and because it later turned out to provide the best extract to inhibit protein aggregation (see below). We decided to sample the leaves of this organism as well as the many other organisms which we later examined, given their easy accessibility and the possibility to carry out uniform sampling for all plants. We acknowledge that leaves constitute a short-lived part of the plant unlike persistent tissue such as the shoot or root apical meristem, making their relevance questionable in terms of longevity studies. However, data from leaves would at worst constitute a false negative, and thus any evidence for anti-aggregative properties even in such ephemeral organs could be considered a robust observation.

**FIGURE 1:**
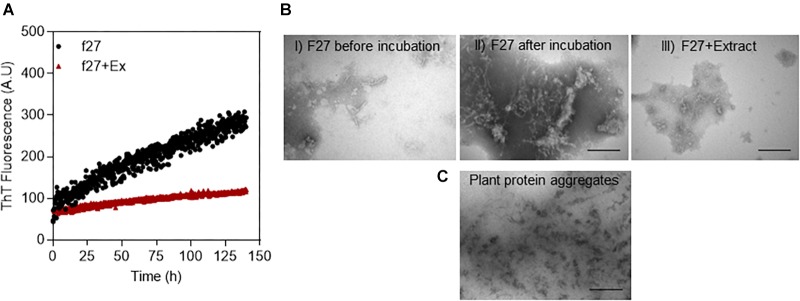
Fibrillation of proteins in extracts from *A. saccharum*. **(A)** Demonstration of fibrillation of f27 proteins (black line) compared to the inhibition of the fibrillation by small molecule extract of *A. saccharum* (red-brown line) through Thioflavin T fluorescence. Soluble proteins extracted from *A. saccharum* were size-fractionated by gel filtration and incubated over 0–5 days. The fibrillation data for the other fractions are shown in Supplementary Figure S2. **(B)** TEM images of f27 proteins recorded under different conditions: before incubation (I) and after 5 days at 37°C without (II) or with small molecule extract of *A. saccharum* (III). Scale bar, 200 nm. **(C)** TEM images of protein aggregates extracted from *A. saccharum* leaves confirm very low amounts of fibrils.

Proteins were crudely fractionated according to size by gel filtration (Supplementary Figure S1A). Partial fractionation was confirmed by concentrating the fractions by ultrafiltration followed by SDS-PAGE analysis (Supplementary Figure S1B). To test for the different fractions’ ability to fibrillate, we used conditions known from other experiments to favor protein aggregation under otherwise mild conditions (neutral pH and 37°C), namely, prolonged and vigorous shaking (Giehm and Otzen, 2010, 2012). The formation of fibrils was followed over time using the compound ThT, whose fluorescence increases significantly when bound to amyloid (Naiki et al., 1989). ThT fluorescence increased for some protein fractions over a 5-day incubation period (Supplementary Figure S2), consistent with general formation of fibrils (data for protein fraction 27 shown in Figure 1A and the remaining fractions in Supplementary Figure S2). There were no fibrils in F27 before extraction according to TEM (Figure 1B, left), but fibril-like structures were observed after 5 days of incubation [Figure 1B(I)]. We then tested whether an unfractionated small-molecule *A. saccharum* extract (here defined as species that could be extracted in acetone, redissolved in water, and passed through a 3 kDa filter) could inhibit this fibrillation. Although the extract was diluted 15-fold, it completely suppressed growth of the ThT signal (Figure 1A), and the absence of fibrils was confirmed by TEM images (Figure 1B, right). Similar results were obtained with *Abies nordmanniana* (Nordmann fir, data not shown).

We also examined *A. saccharum*’s population of protein aggregates (i.e., proteins not solubilized in the normal extraction process) for the possible presence of fibrils. The TEM image shows amorphous aggregates but no fibrils (Figure 1C) and the ThT showed no fluorescence intensity when it was added to the aggregates (data not shown).

### Acetone Was Selected as the Best Solvent to Extract Small Molecules From the Plant Leaves

**FIGURE 2:**
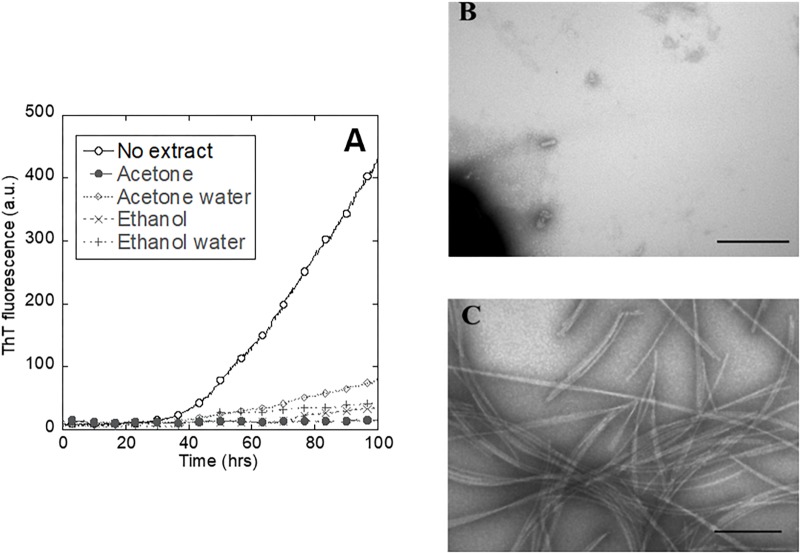
Inhibition of αSN fibrillation by different small-molecule extracts from *A. saccharum.* **(A)** αSN fibrillation assessed by thioflavin T fluorescence during prolonged incubation in the absence and presence of extracts made using 100% acetone, 80% acetone – 20% water, 100% ethanol, and 50% ethanol – 50% water. TEM images of αSN samples incubated **(B)** in the presence of acetone extract and **(C)** just in buffer confirm that the plant extract inhibits protein fibrillation. Scale bar: 200 nm.

To select the best solvent to extract small molecules from the plant leaves, we tested the inhibitory effect of extracts obtained with different solvents on the aggregation of the amyloidogenic protein αSN, whose aggregation is central to the development of Parkinson’s disease (Volles and Lansbury, 2003). The extract made with 100% acetone turned out to completely suppress the ThT signal (Figure 2A) and was also accompanied by a complete lack of fibrils according to TEM (Figure 2B). In contrast, αSN incubated without extract produced a large quantity of straight unbranched fibrils (Figure 2C). We found that the plant extract causes some ThT signal quenching at the used concentration (data not shown). However, while the absolute values of the ThT signal cannot be used as a quantitative measure of the extent of fibrillation, it is reasonable to use changes in the time course of ThT fluorescence as an indication of interference with fibrillation. Accordingly, further analysis of fibrillation based on ThT fluorescence was based on the lag time preceding growth of ThT fluorescence.

### Ranking of Broad Ranges of Plant Samples Based on Their Potential to Inhibit αSN Fibrillation

**FIGURE 3:**
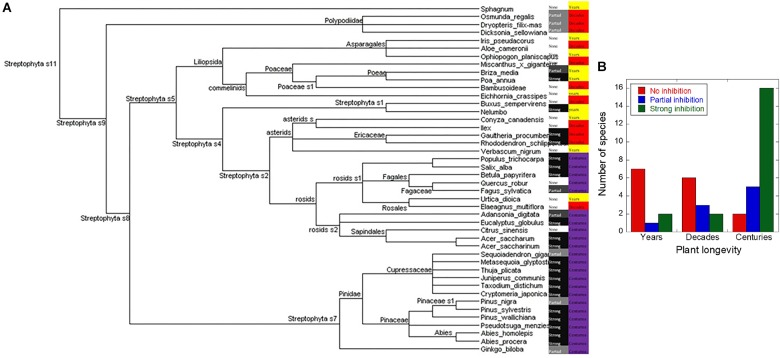
Correlation between plant longevity and ability to inhibit fibrillation. **(A)** Evolutionary relationships between the screened plants as indicated by the NCBI phylogeny database with family indicated. Subcategories are indicated only when they are needed to categorize the tested plants as belonging to the same group, for example the subgroups for *sphagnum* are not shown because its only point of commonality with the other plants belongs to Steptophyta s11. Plants yielding extracts that were strongly inhibitory are indicated in black, partially inhibitory in gray, and those having no effect in white. Each plant’s maximum attainable age is listed as either years (yellow), decades (red), or centuries (purple). **(B)** The data from **A** illustrated as a bar graph, showing the number of plant showing no inhibition (red), partial inhibition (blue), and strong inhibition (green) for plants that live for years, decades, and centuries, respectively.

To investigate the inhibitory effect of extracts across a broad range of plant samples, we prepared extracts from 29 different plants spanning a large part of the plant kingdom and available from the Aarhus Botanical Gardens (Figure 3A). Samples were obtained from approximately the same wet mass of plant material (typically leaf tissue), making it possible to make a rough comparison based on the biological concentration of these materials; 10 μl of these extracts were then tested for their ability to inhibit aggregation of αSN in ThT plate reader assays. Increasing the volume to 100 μl led to a zero ThT signal in all samples tested, but EM imaging showed that fibrils were still present in similar amounts to the control for the samples previously characterized as non-inhibitors, though they were absent from those characterized as partial inhibitors (data not shown). Therefore, we limit ourselves to analysis at low extract concentrations.

Some of the extracts caused complete inhibition of fibrillation over a 48-h period (as verified by EM, data not shown), and other caused significantly increased lag times (see below). We carried out additional sampling from the more promising subgroups, and added more control samples, to bring the total number of sampled plants up to 49 (Supplementary Table S1). For practical reasons (season-dependent harvesting of samples), several analyses were limited to the original 29 samples. There is a strong correlation between the maximum attainable age for the plant and the inhibitory effects (Figure 3A). This is quantified in Figure 3B which shows that only 30% (3 out of 10) of the plants that live less than a decade had an anti-fibrillatory effect; this number rises to 45% (5 out of 11) for those that live for decades, and 91% (21 out of 23) for those that live for centuries. The percentages showing strong inhibition were 20, 18, and 70% for years, decades, and centuries, respectively, also emphasizing the superior performance of long-lived plants. Partial inhibition was most common in decades old plants (27.2%) followed by centuries-old plants (22%) and least prevalent among the plants that only live for years (10%).

**FIGURE 4:**
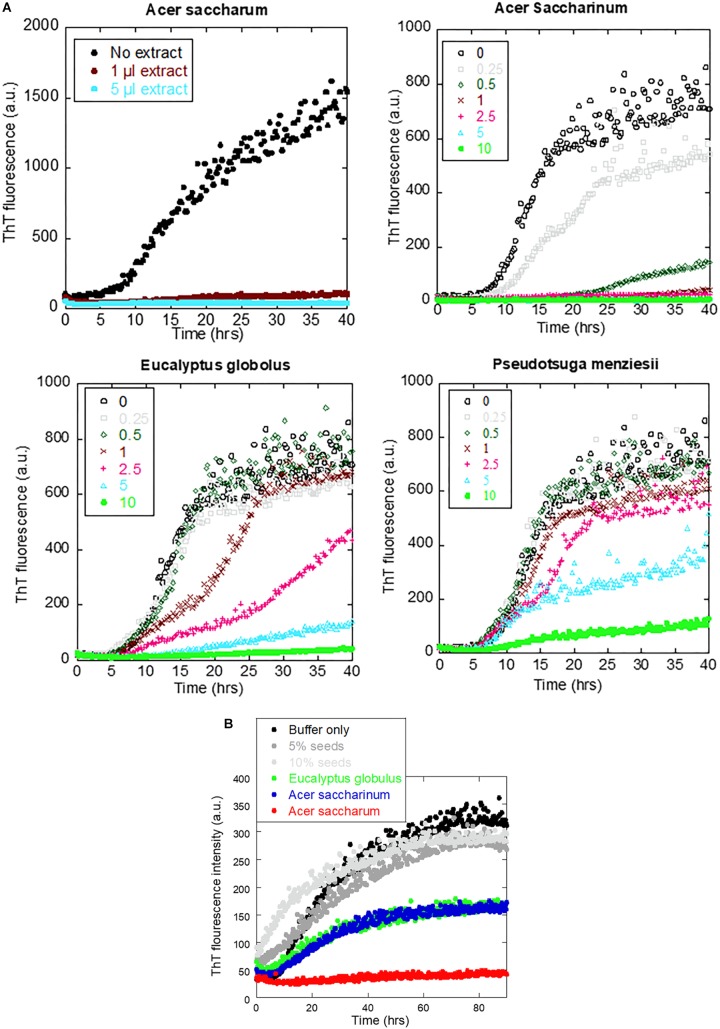
Effect of extracts from the four indicated plants on αSN fibrillation. **(A)** Fibrillation of 1 mg/ml αSN in the presence of 0–10 μl of extracts and **(B)** fibrillation of 1 mg/ml monomeric αSN fibrillation alone (black), in the presence of 5 or 10% pre-existing seeds (gray) or in the presence of 5% seed and 1 μl plant extract (colored points). Incubation at 37°C in 100 mM Tris pH 8.5 with one glass bead, 12-min cycles including 10-min orbital shaking followed by reading of fluorescence.

To identify which extracts were the most efficient, we carried out αSN fibrillation assays for dilutions of the four extracts that showed no fibrillation according to EM or ThT fluorescence. Extracts were dried and weighed to be able to rank them in terms of effect per weight (Table 1). We found that *A. saccharum* was the most efficient, followed by *A. saccharinum*. The next two on the list, *E. globolus* and *P. menziesii*, were both significantly less efficient. Time courses for the effect of these extracts on αSN fibrillation are shown in Figure 4A. For comparative purposes, we include the amount of material required to cause complete inhibition of the ThT fluorescence signal relative to that of EGCG, one of the most efficient general inhibitors of fibrillation (Ehrnhoefer et al., 2008). Remarkably, the two *Acer* extracts are significantly more efficient than EGCG on a per-mass basis, in contrast to *E. globolus* and *P. menziesii*.

The ability of the extracts of *A. saccharum, A. saccharinum*, and *E. globolus* to prevent the seeding of αSN fibrillation by sonicated fibrils was tested by adding plant extract to the seeds and incubating for 24 h before adding them to the monomer (to provide time for possible dissociation or remodeling of the seeds). Addition of seeds to monomeric αSN without any extracts abolished the ∼8-h lag phase of fibrillation as expected (Figure 4B); extracts from *A. saccharinum* and *E. globolus* restored the 8-h lag phase and reduced the final ThT level, while *A. saccharum* extract completely suppressed fibrillation, emphasizing its preeminence as aggregation inhibitor.

**FIGURE 5:**
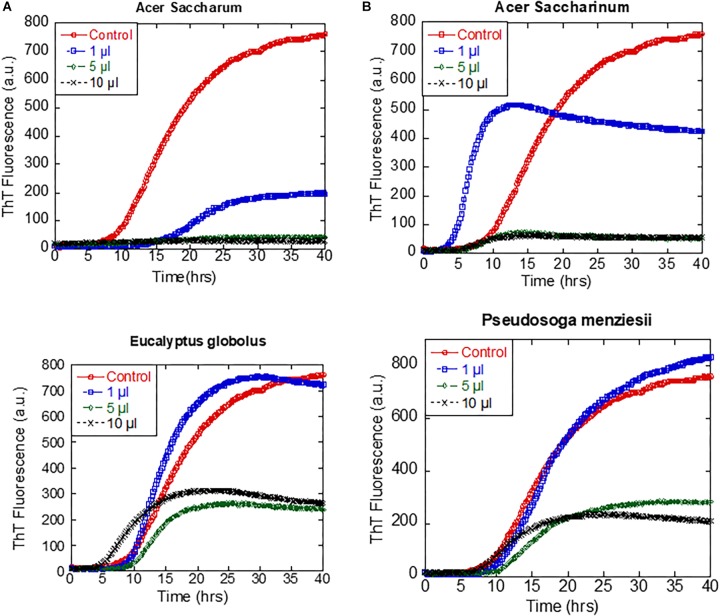
**(A)** Inhibition of fibrillation of 40 μM Aβ by 0–10 μl of extracts and **(B)** 10 μM amylin by 1 μl of extracts from the four indicated plants. In both cases, incubation at 37°C in 100 mM Tris pH 8.5 without shaking and reading every 10 min.

To examine whether these inhibitory effects extended to other aggregating proteins and peptides, we turned to the Aβ40 peptide involved in Alzheimer’s disease. We found inhibitory effects for all of the highly effective αSN fibrillation inhibitors (Figure 5A). Both *A. sacharum* and *A. saccharinum* totally inhibited Aβ40 fibrillation at 10 μL per well, extracts of *P. menziesii* caused delayed and decreased fibrillation and *E. globolus* extract only reduced ThT fluorescence end point levels but with the same lag time. Further studies with *P. menziesii* were discontinued because of relatively weak effects compared to the *Acer* species and limited availability. For additional support, we examined the amyloidogenic peptide amylin involved in type 2 diabetes. Also here, as little as 1 μl of both *Acer* species and *E. globolus* extracts were all very efficient at reducing amylin fibrillation as followed by ThT-fluorescence (Figure 5B).

### Identification of Specific Inhibitory Compounds by Fractionation

**FIGURE 6:**
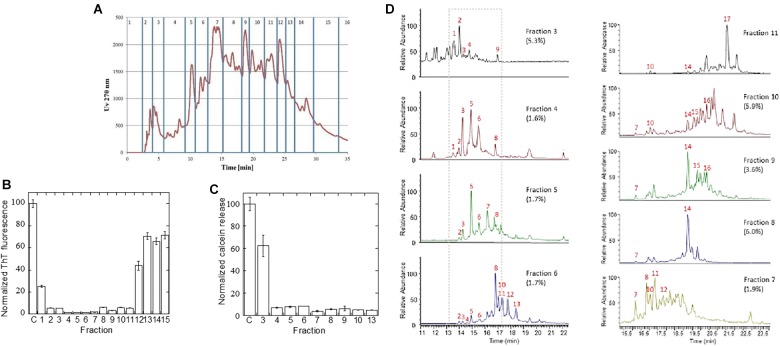
Fractionation of *A. saccharum* extract to identify inhibitors. **(A)** UV absorbance of the reverse phase fractionation over a Jupiter C18 RP-HPLC column of the small molecule extract in red, with fractions 1–16 indicated in blue. **(B)** Normalized ThT fluorescence of αSN fibrillation in the presence of the fractions defined in Figure 5A based on raw data shown in Supplementary Figure S3. **(C)** Membrane permeability assay: Normalized release of calcein by 2 μM αSN oligomers with or without pre-treatment with 5 μL extract equivalent of fractionated *A. saccharum* extract. All extracts have been verified not to cause calcein release without oligomer (not shown). **(D)** Total ion chromatograms from LC-MS (negative ion mode A) chromatography used to identify active components within inhibitory fractions. Numbers refer to compounds in Supplementary Table S2.

The *A. saccharum* extract showed the most pronounced inhibitory effect on fibrillation of both αSN, Aβ, and amylin. To identify which compounds were responsible for this, the extract was fractionated by reverse phase HPLC on a Jupiter C18 column (Figure 6A), and the 15 resultant fractions were individually tested in an αSN fibrillation assay (Supplementary Figure S3). The total level of ThT fluorescence was dramatically decreased in the presence of several of these compounds (summarized in Figure 6B), particularly fractions 2–11. To provide a complementary analysis of the compounds’ effects, we measured their ability to prevent or reduce the αSN oligomer’s permeabilization of phospholipid vesicles through release of calcein. The αSN oligomer is known to perturb anionic phospholipid vesicles, allowing efflux of small molecules trapped within the vesicle; aggregation inhibitors such as EGCG are able to prevent this by binding to the oligomer and reducing its membrane affinity (Lorenzen et al., 2014b). In this assay, particularly fractions 4–10 as well as fraction 13 showed remarkable efficiency (Figure 6C), giving reasonable though not complete overlap with ThT data.

Finally, we used high-resolution UHPLC-MS in negative ion mode to identify individual compounds in the individual *A. saccharum* fractions. This revealed that each of the analyzed fractions contained multiple compounds. MS-MS analysis and comparison of retention times to authentic standards were used in combination for structural determination (Supplementary Table S2). For fractions 3–6, the total ion chromatograms (Figure 6D) included major peaks for a range of known compounds including catechin and epicatechin ([M-H]^-^ at *m/z* 289, C_15_H_13_O_6_) appearing at 14.28 and 16.8 min, respectively, and dimeric catechin procyanidin B isomers ([M-H]^-^ at *m/z* 577, C_30_H_25_O_12_) which were characterized by an MS-MS fragment at *m/z* 289. Peaks 4 and 10 (*m/z* 633) corresponded to metabolites with a molecular formula of C_27_H_22_O_18_ and MSMS fragmentation displayed fragment ions (*m/z* 463, 301, and 169) suggestive of isomers of galloyl-hexahydroxydiphenoyl-glucose. This is a well-known ellagitannin found in numerous plant species (Iranzo, 2011) including *A. saccharum.* The fragment *m/z* 463 relates to M-H-galloyl while fragments at *m/z* 301 and 169 relate to ellagic acid and galloyl entities, respectively. MS-MS fragmentation patterns of peak 9 (*m/z* 647) included an additional ion at *m/z* 433 having a formula of C_19_H_13_O_12_ which was indicative of an ellagic acid xyloside derivative. Peaks 11 (*m/z* 517.5) and 13 (*m/z* 516.5) were doubly charged and corresponded to compounds with molecular weights of 1,036 and 1,034, respectively. The identity of these peaks could not be determined although MS-MS spectra of these ions both included an ion at *m/z* 169 which corresponded to the known gallic acid fragment (C_7_H_5_O_5_), further indicating that a range of galloylated derivatives were present across the fractions, the majority appearing in fraction 6. Unlike other entities which appeared in specific fractions, the known fibrillation inhibitor chlorogenic acid [23,24] (with [M-H]^-^ at *m/z* 353, C_16_H_17_O_9_) appeared in each chromatogram at 14.9 min with highest levels in fractions 4 and 5. This metabolite was assigned by a strong MS-MS fragment at *m/z* 191 (C_7_H_11_O_6_) relating to the quinate moiety and *via* comparison of its retention time to that of an authentic standard.

Although several of the inhibitory fractions contained chlorogenic acid, fractions 7–11 contain no chlorogenic acid, yet still show strong inhibitory effects (Figure 6B). Analysis of the LC-MS data of these fractions showed that there were no major metabolite peaks that were consistently present in all of these fractions (Figure 6B). While the total ion chromatogram of fraction 7 contained peaks which overlapped with those of fraction 6, fraction 8 and 9 were dominated by a peak with *m/z* at 468 (19.25 min). This ion was again doubly charged and related to a molecule with a formula of C_41_H_30_O_26_, the [M-H]^-^ ion at *m/z* 937 being clearly visible in the MS spectrum. The identity of this peak was assigned to a trigalloyl-hexahydroxydiphenoyl-glucose derivative such as Davidiin, an ellagitannin previously reported to be present in *A.* *saccharum* (Hatano et al., 1990). Fractions 10 and 11 also each contained differential peaks to those of earlier fractions with fraction 11 being dominated by pentagalloyl glucose appearing at 21.55 min.

### Effect of Identified Plant Compounds on αSN Aggregation

Based on the LC-MS data in combination with the known literature, we selected five compounds (procyanidin B2, pentagalloylglucose, ellagic acid, chlorogenic acid, and catechin) for more detailed studies on their effects on αSN structure and aggregation. We started by interrogating the compounds’ ability to affect the interaction of monomeric αSN with DMPG vesicles. When αSN is added to DMPG vesicles, it undergoes a transition to a structure rich in α-helical structure. When each of the small molecules (40 μM) was titrated into a solution of αSN and DMPG, this helical structure is maintained (Supplementary Figure S4), suggesting that the compounds cannot displace the protein from the lipid membranes and reduce the α-helical content of αSN. Thus, they do not directly reduce monomeric αSN’s interaction with membranes.

**FIGURE 7:**
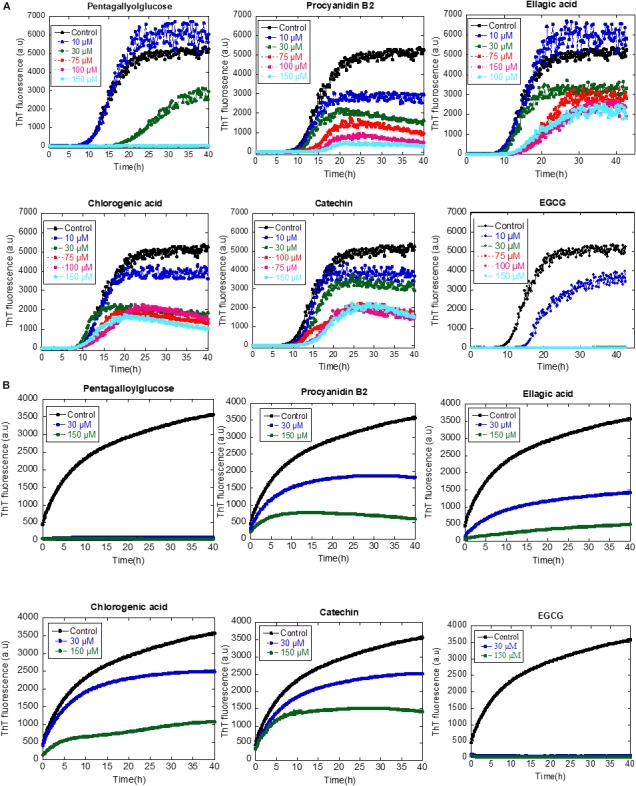
**(A)** The effect of the identified compounds (10–150 μM) on αSN fibrillation. **(B)** The effect of the identified compounds (30 and 150 μM) on the seeding of αSN aggregation.

Subsequently, we addressed the inhibitory effect of 10–150 μM of the compounds on aggregation of αSN as monitored by a ThT-binding assay. All compounds had an inhibitory effect, but they varied significantly in their potency; only pentagalloylglucose inhibited fibrillation completely at 75 μM, similar to the internal control EGCG, whereas the rest of the compounds only inhibit weakly (Figure 7A).

To determine whether or not the compounds can affect the secondary pathway of αSN, we used seeding to bypass the primary nucleation, adding short sonicated fibrils to monomeric αSN to accelerate the nucleation phase of amyloid formation. In all cases, 30 and 150 μM of the tested compounds inhibited fibril formation. Notably, the inhibitory effect of pentagalloylglucose was higher than other compounds and comparable to EGCG (Figure 7B).

**FIGURE 8:**
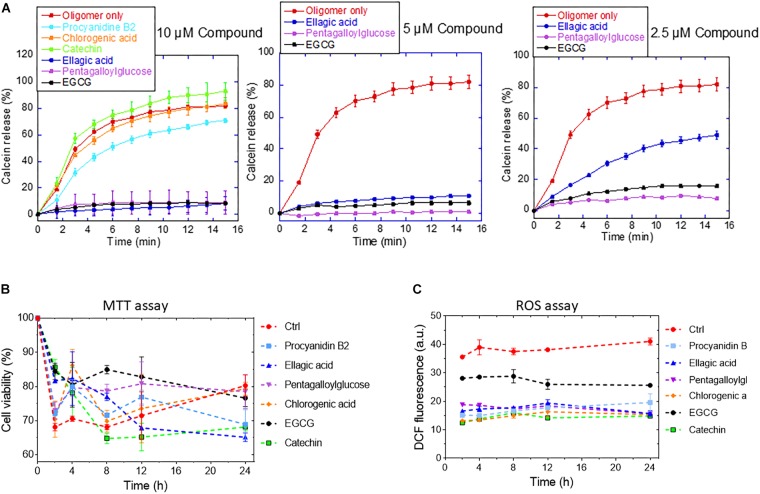
**(A)** Calcein release from DOPG vesicles in the presence of 2.5–10 μM of the different compounds. **(B)** Viability (MTT assay) and **(C)** ROS production of OLN-93 cells after 24 incubation with αSN aggregates formed alone and in the presence of the compounds.

Next, we examined the ability of the compounds to change the membrane permeabilization by αSN oligomers using calcein-loaded DOPG vesicles. The permeabilization of phospholipid membranes by αSN oligomers may be considered a simple proxy for cellular toxicity (Lorenzen et al., 2014b). Oligomers were pre-incubated with compounds and then added to the vesicles. Oligomer-induced calcein release was significantly inhibited by pentagalloylglucose and EGCG even at 0.5 μM, but only weakly inhibited by the rest of the compounds (Figure 8A).

To determine the effect of the compounds on the formation of toxic aggregates during the fibrillation process over different times (0–24 h), we conducted an MTT and a ROS assay with OLN-93 cells. The MTT assay (Figure 8B) showed that αSN aged in the absence of the compounds decreased cell viability up to 8 h, after which the deleterious effect on cell viability decreased, consistent with the view that cytotoxic oligomers accumulate in the early stages of protein aggregation (Bucciantini et al., 2002). In contrast, aggregates resulting from the incubation of αSN with EGCG, procyanidin B2, pentagalloylglucose, and chlorogenic acid were significantly less toxic. The decreased toxicity could be due to the removal of toxic aggregates and their conversion to other aggregates or a reduction of their ability to bind to cellular membranes as suggested by the calcein-release experiments (Figure 8A).

To evaluate the ROS production values in OLN-93 cells, the cells were exposed to αSN aggregates formed with and without compounds. We saw a significant decrease in the level of ROS production of cells exposed to the aggregates of αSN incubated with the compounds at different times (Figure 8B). Interestingly, the level of ROS production in the presence of the aggregates remained essentially constant throughout the aggregation process.

## Discussion

This study has attempted to link plant longevity with the presence of anti-aggregative compounds. We would like to make the important disclaimer that we do not consider the ability of small molecule plant metabolites to inhibit unwanted protein aggregation to be the predominant evolutionary driving force in the development and regulation of these compounds. Plant longevity is determined by an enormous array of different factors which will contribute to different extents, and the small molecules we have identified as inhibitors serve a multitude of different functional purposes in plant physiology, many of which are unrelated to aggregation. It is also natural to expect that plant proteins have been selected both for their biological function and for their ability to avoid unwanted aggregation, as is known in proteins from many other organisms, cfr. gatekeeper residues (Otzen et al., 2000; Rousseau et al., 2006). Furthermore, our approach does not do justice to compartmentalization and distribution of small molecules and proteins within the cell which is lost upon extraction and is not recreated in our very simplistic inhibition assays. It could, however, be hypothesized that it would be useful for plants to produce and maintain small molecules which in addition to their intrinsic metabolic functions also suppress unwanted aggregation of proteins, and that such properties could contribute (in, however, modest a way) to the extension of plant life times. Strong proof of a causal relationship between anti-aggregative properties and longevity is very difficult to establish and is far beyond the scope of the present study, but our rather naïve attempts to establish a correlation between anti-aggregative properties and longevity are not inconsistent with such a relationship. At the very least, our observations constitute an empirical basis for more systematic attempts to go treasure-hunting in the vast collection of natural plant compounds for compounds against aggregation-driven neurodegenerative diseases.

With these caveats in mind, we cautiously speculate on the possible role of anti-aggregators in plants. At the start of this project, we considered two reasons to explain the absence of endogenous plant protein fibrils. Either the proteins have low intrinsic fibrillation propensities or there is a mechanism for inhibiting fibrillation. We tested the intrinsic fibrillation propensity by purifying and incubating soluble plant proteins, and found that multiple semi-purified *A. saccharum* protein fractions fibrillate quite readily upon shaking, ruling out intrinsic low fibrillation propensity. This left fibrillation inhibition as the most likely explanation (ruling out less likely mechanisms such as a plant pathogen response or the presence of physical barriers like the cell wall). Therefore, we added *A. saccharum* small molecules back to the protein mix and found that this efficiently inhibited fibrillation. It is also clear that we have not caused conditions where all the proteins fibrillate, as many other structures are present in the EM pictures. The fact that the same small molecules extracts inhibit the fibrillation of unrelated pathogenic proteins such as αSN, Aβ40, and amylin is strong evidence that these small molecules are key to the absence of protein fibrillation in *Acer saccharum*.

From here, the target was to show if the ability to inhibit fibrillation was broadly distributed in the plant kingdom, and we therefore extracted small molecules from a broad range of plants. Here, we observed that inhibitory effect are generally stronger for the longer lived plants, though there are both long-lived plants which do not function as inhibitors, and short-lived plants which function well. We do, however, see strong inhibitors in all the branches of the tree of life, though the age connection seems weaker in the grasses. This could be because grasses in general senesce their flowering stems at the end of the growing season, and therefore while the plant may live for decades, the individual biomass does not, and inhibition may therefore not be required. In future work, this could potentially be tested by including palm trees which are both monocots and have long living persistent tissue. It may appear that the conifers have a better inhibition than other branches, though this may also reflect the fact that the conifers are all long lived. Disregarding conifers, we still find 55% strong inhibitors, 27% partial inhibitors, and 18% non-inhibitors for the century old plants, whereas 70% of the plants that live less than a decade and 54.54% of those that live for decades have no measurable inhibitory effect. This means that we see a stronger inhibitory effect for the longer lived plants, which suggests that this is an effect that is selected for as plants grow older. Nevertheless, this raises the question why leaves from some long-lived plants such as *Citrus sinensis* (orange) and *Quercus robur* (oak) do not show inhibitory activity? In the absence of concrete data, we speculate that there is a higher concentration in longer lived tissue; oak loses its leaves each year and citrus trees are prone to large scale leaf loss under a number of conditions; in other words, leaf extracts could constitute false negatives because they are not representative of the entire physiology of the organisms. Added to this the concentration in the actual tissue is at least 15-fold higher than in our assay, so it is entirely possible that there is an inhibitory effect at physiological concentrations.

From an evolutionary standpoint, it is very interesting that a number of short lived plants inhibit protein fibrillation. This suggests both that fibrillation inhibition is endemically present in nearly all plants, but not present in effective concentrations in those plants where evolutionary pressure does not select. Note also that the inhibitors already identified also have different metabolic functions. Thus, EGCG is a cell wall regulating component (Lewis et al., 2008), while chlorogenic acid, an aggregation inhibitor identified both here and elsewhere (Cheng et al., 2011; Wei et al., 2016) is part of the lignin metabolism (Taylor and Zucker, 1966). In many cases, only a few mutations can be required for an organism to increase the amount of an existing metabolite molecule (e.g., a single point mutation in the promotor of a single gene coding for a biosynthetic enzyme). Given the variety of plant metabolites and biosynthetic pathways, it could be imagined that a plant species in this way could frequently accumulate mutations which would mitigate protein fibrillation and promote longevity. The mutations would then be retained, provided they had no serious negative repercussions.

## Conclusion

The fibrillation of native proteins has not been observed in plants; here we show that this is due to the presence of fibrillation inhibitors in nearly all plants that live longer than a century. This inhibitory effect is the result of numerous different small molecules. These cocktails of small molecules contain known inhibitors, and are by weight more efficient than most of the known fibrillation inhibitors. The broad range of extracts found during our screening, and the remarkable success rate (91% at least partial inhibitors for plants with centuries-long lifespans) indicate that there should be great potential for identification of non-toxic effective inhibitors from the plant kingdom.

## Data Availability

All datasets generated for this study are included in the manuscript and the Supplementary Files.

## Author Contributions

HM-B conducted the experiments, and wrote the manuscript. LK developed concepts, conducted experiments, and wrote the manuscript. HE, FA, GC, and GR conducted the experiments. JW conducted the experiments, analyzed the data, and wrote the manuscript. DO developed concepts, designed the experiments, analyzed the data, provided funding, and wrote and edited the manuscript.

## Conflict of Interest Statement

The authors declare that the research was conducted in the absence of any commercial or financial relationships that could be construed as a potential conflict of interest.

## Abbreviations

αSN: α-synuclein
DCFH-DA: 2′,7′-dichlorodihydrofluorescein diacetate
DMPG: 1,2-dimyristoyl-sn-glycero-3-[phospho-rac-(1-glycerol)]
DOPG: 1,2-dioleoyl-sn-glycero-3-[phospho-rac-(1-glycerol)]
EGCG: epi-gallocatechin gallate
MTT: 3-[4,5-dimethylthiazol-2-yl]-2, 5-diphenyltetrazolium bromide
TEM: transmission electron microscopy
ThT: thioflavin T.
