# Supplementary material for: A Possible Connection Between Plant Longevity and the Absence of Protein Fibrillation: Basis for Identifying Aggregation Inhibitors in Plants
Source: Front Plant Sci. 2019 Feb 13;10:148. doi: 10.3389/fpls.2019.00148 (PMC6381023; doi:10.3389/fpls.2019.00148)
Supplement: Supplementary file 1 [file Data_Sheet_1.pdf]

A possible connection between plant longevity and the absence of protein fibrillation: Identifying aggregation inhibitors in plants

Hossein Mohammad-Beigi, Lars Kjaer, Hoda Eskandari, Farhang Aliakbari, Gunna Christiansen, Gianluca Ruvo, Jane L. Ward, Daniel Otzen

Supplementary Information

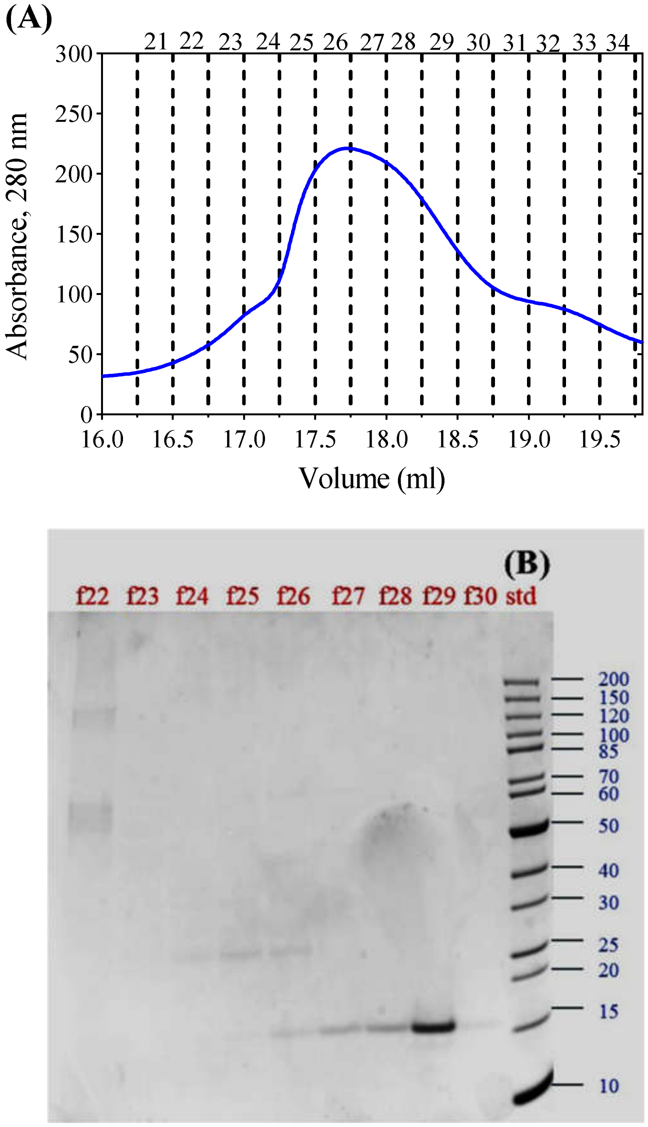

Fig. S1. (A) Fractionation of *A.saccharum* extract proteins on a 24 mL Superose 6 column. (B) Fractions of 0.25 ml were collected, concentrated with 3 kDa cut-off Millipore spin filters, and run on SDS-PAGE.

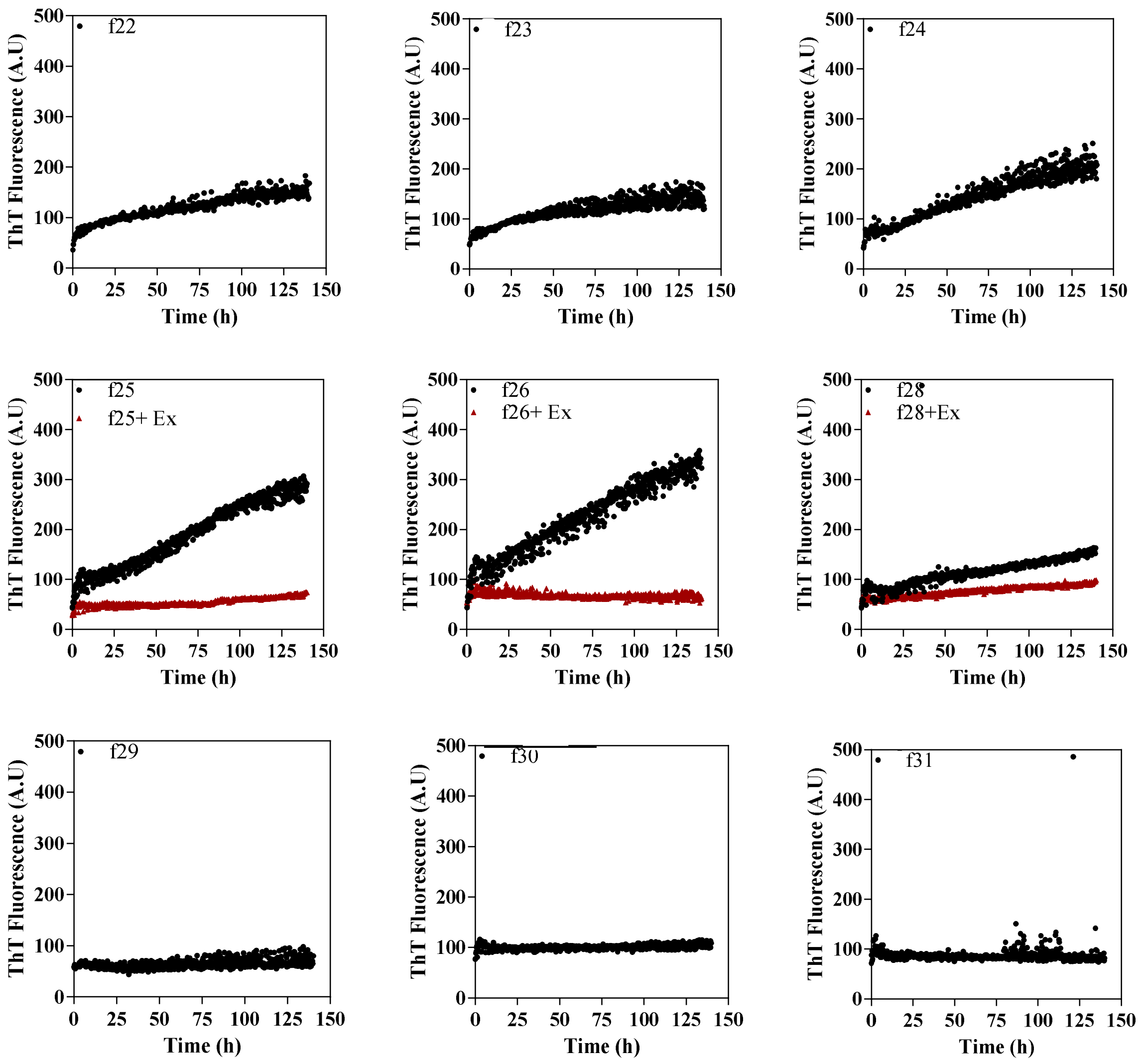

Fig. S2. Fibrillation of proteins in extracts from *A. saccharum*. Soluble proteins extracted from *A. saccharum* were size-fractionated by gel filtration and incubated at 37ºC in 100 mM Tris pH 8.5 at 1 mg/ml protein either alone (black data points) or with small-molecule extract from *A. saccharum* ("+ Ex", red-brown color).

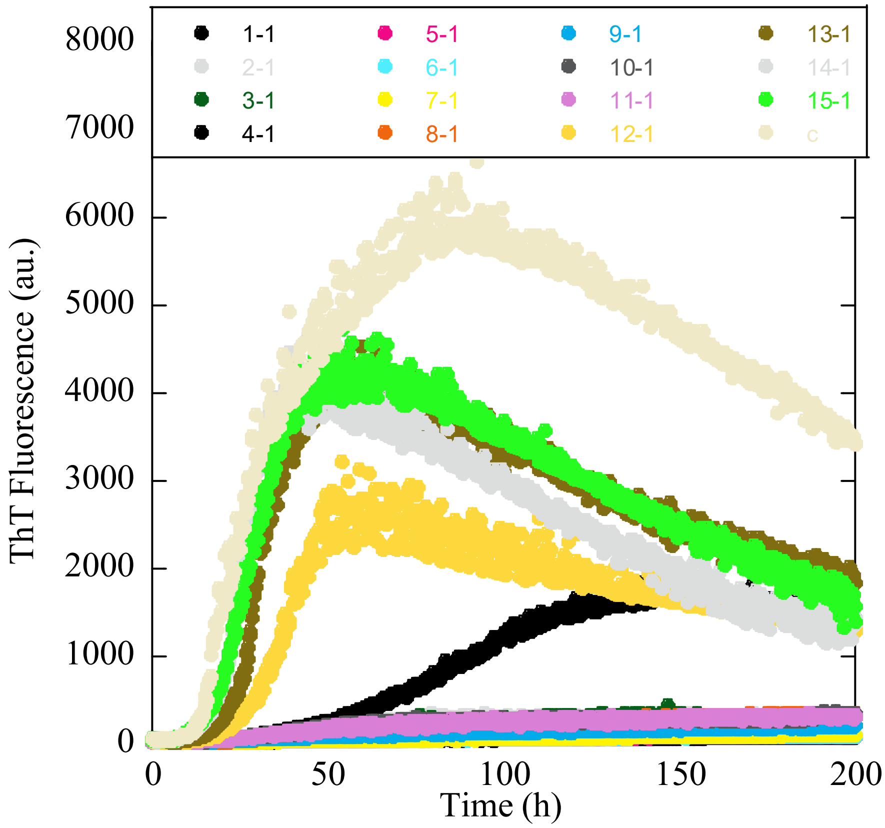

Fig. S3. Time course for fibrillation of αSN in the presence of 10 µl of fractions of *Acer saccharum* extract fractionated on a Jupiter C18 column.

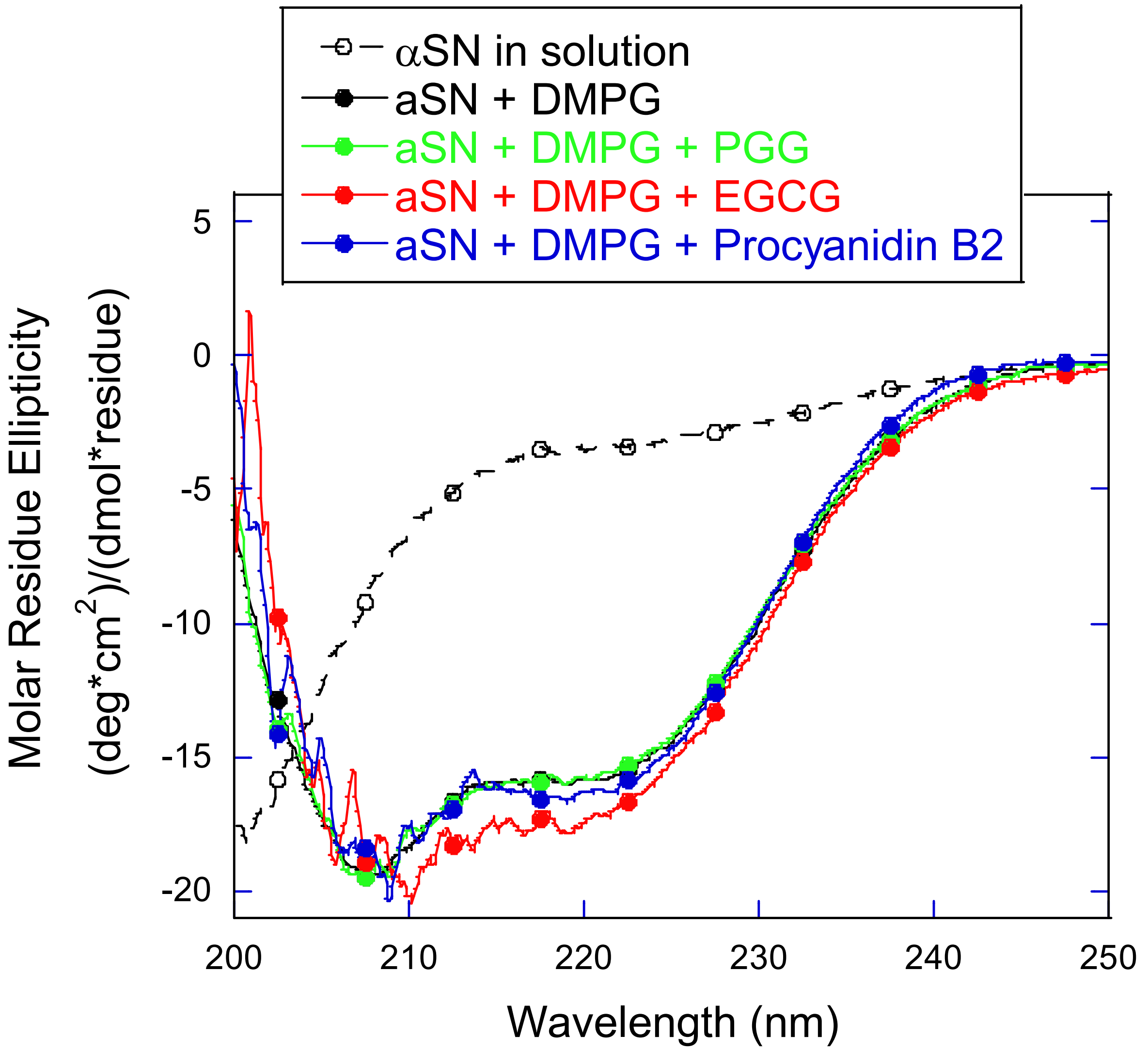

Fig. S4. CD spectrum of αSN (14 μM) either alone in solution or in the presence of DMPG vesicles (0.2 mg/ml) with or without 40 μM of small molecule inhibitors.

Table. S1. List of plant samples collected from Aarhus Botanical Garden

No	Scientific name	Common name	Age (range)	Collection	Inhibitory effect	Ref
1	*Verbascum nigrum*	Dark Mullein	Annual	April 2013	none	1
2	*Ophiopogon planiscapus "Niger"*	mondo grass	Annual, perennial grass	April 2013	none	2
3	*Conyza canadensis*	Canada Fleabane	annual	April 2013	none	1
4	*Briza media*	common quaking grass	Annual, perenial	April 2013	partial	2
5	*Fagus sylvatica*	Europea Beech	Hundreds of years	April 2013	strong	1
6	*Datura*	Thorn appel	Annual	April 2013	None	1
7	*Pinus nigra*	Black Pine	Hundreds of years	April 2013	Partial, high dose give s total inhibition	1
8	*Acer saccharinum*	Silver Maple	Hundreds of years	April 2013, September 2013, April 2014, September 2014. Same results with same dosage,	Strong	1
9	*Poa Annua*	Annual meadow grass	Annual	April 2013	Strong	1
10	*Dryopteris filix-mas*	Male evergreen fern	decades	April 2013	Partial	1,2
11	*Osmunda Regalis*	Royal Fern	decades	April 2013	Partial	1
12	*Pseudotsuga menziesii*	Douglas Fir	Hundreds of years	April 2013	Strong	1
13	*Sequoiadendron giganteum*	Giant Redwood	Thousand plus years	April 2013	Partial	1
14	Quercus robur Festigiaia	English Oak	Centuries	April 2013	none	1
15	*Metasequoia glyptostroboides*	Dawn Redwood	Hundreds of years	April 2013	Strong	1
16	*Ilex*	Holly	Decades	April 2013	none	1
17	*Miscanthus Giganteus*	Giant Miscanthus	Decades (root biomass only perennial)	April 2013	none	1
18	*Buxus sempervirens*	Common box	Decades	April 2013	none	1

19	*Iris pseudacorus*	Paleyellow iris	Annual	April 2013	none	1
No	Scientific name	Common name	Age (range)	Collection	Inhibitory effect	Ref
20	*Urtica dioica*	Stinging Nettle	Annual (perennial)	April 2013	none	1
21	*Dicksonia Sellowiana*	None	decades	April 2013	none	2
22	*Eucalyptus globulus*	Tasmanian Blue Gum	Centuries	April 2013	strong	1
23	*citrus sinensis*	Sweet orange	Century	April 2013	none	1
24	*Sphagnum*	Sphagnum Moss	Annual/hard to determine	April 2013	none	1
25	*bambusa vulgaris*	Giant Bamboo	Decades-above ground Annual root biomass decades	April 2013	none	2
26	*adansonia Digttata*	Judas Fruit	Hundreds of years	April 2013	partial	1
27	*Eichhornia crassipes*	Water Hyacinth	Annual (perennial)	April 2013	none	1
28	*Nelumbo*	Sacred water lotus	Annual (perennial)	April 2013	Strong	1
29	*Pinus sylvestris*	Scots pine	centuries	August 2013	strong	
30	*Rhododendron schlippenbachii*	royal azalea	decades	August 2013	strong	2
31	*Abies procera*	Abies procera	centuries	August 2013	Strong	1
32	*Gaultheria procumbens*	Eastern teaberry	Decades	August 2013	Strong	1
33	*abies homolepis*	Nikko Fir	centuries	August 2013	Strong	1
34	*Aloe Cameronii*	Aloe vera	Decades	April 2013	none	3
35	*Abies procera*	Noble Fir	Centuries	August 2013	Strong	1
36	*Gaulteria procumbens*	Eastern teaberry	Centuries	August 2013	Strong	1
37	*abies homolepis*	Nikko Fir	Centuries	August 2013	Strong	1
38	*Ginkgo biloba*	Maidenhair Tree	Centuries	August 2013	Strong	1
39	*Taxodium Dissectum*	Bald cypress	Centuries	August 2013	Strong	4
40	*Acer saccharum*	Sugar Maple	Centuries	August 2013, April 2014	Strong	1
41	*Cryptomeria japonica Cristata*	Japanese Cedar	Centuries	August 2013	Strong	1

42	*Pinus wallichiana*	Himalayan Blue Pine	Centuries	August 2013	Strong	1
43	*Juniperus communis*	Common Juniper	Centuries	August 2013	Strong	1
No	Scientific name	Common name	Age (range)	Collection	Inhibitory effect	Ref
44	*Thuja plicata*	Western red cedar	Centuries	August 2013	Strong	1
45	*Betula papyrifera*	Paper Birch	Centuries	August 2013	Strong	1
46	*Salix alba 'Tristis'*	White Willow	Centuries	August 2013	Strong	1
47	*Populus triocarpa*	Black Cottonwood	Centuries	August 2013	Strong	1
48	*Juglans regia*	Walnut	Centuries	August 2013	Strong	3
49	*Elaeagnus multiflora*	Cherry silverberry	Decades	August 2013	none	1,3

Notes:

1) http://www.pfaf.org

2) http://www.missouribotanicalgarden.org

3) J. Fernandez-Lopez, N. Aleta, R. Alia, Noble Hardwoods Network: Report of the Fourth Meeting, 4-6 September 1999, Gmunden, Austria and the Fifth Meeting, 17-19 May 2001, Blessington, Ireland, J. Turok, G. Eriksson, K. Russel, S. Borelli, eds. (Bioversity International, 2002), pp. 38–43.

3) Earle, Christopher J., ed. (2018). "Taxodium distichum subsp. distichum". The Gymnosperm Database.

Table. S2. MS-MS analysis of the compounds identified in fractions 3-11 from an *A. saccharum* extract fractionated over a Jupiter C18 RP-HPLC column. Peaks refer to chromatographic peaks in Fig. 6D.

Peak[a]	Retention time (min)	m/z	Predicted ion formula	Delta[b] (mmu)	MS-MS	λ_{max} (nm)	Identity/ Compound class
Fractions 3-6 (Fig. 7A)							
1	13.59	577.13456	$[C_{30}H_{25}O_{12}]^-$	-0.39	451, 425, 407, 289, 245, 161, 125	280	Procyanidin B1
2	13.98	577.13464	$[C_{30}H_{25}O_{12}]^-$	0.11	451, 425, 407, 289, 245, 161, 125	280	Procyanidin B3
3	14.28	289.07214	$[C_{15}H_{13}O_6]^-$	0.35	245, 205, 179, 125	280	Catechin
4	14.78	633.07290,<br>483.07895,<br>465.10392	$[C_{27}H_{21}O_{18}]^-$<br>$[C_{20}H_{19}O_{14}]^-$<br>$[C_{21}H_{21}O_{12}]^-$	0.7<br>1.1<br>1.2	463, 301, 275, 169<br>301, 169, 125<br>285, 259, 193, 125	279	Galloyl-HHDP[c]-glucose
5	14.94	353.08759	$[C_{16}H_{17}O_9]^-$	-0.16	191, 179	288, 329	Chlorogenic acid
6	15.51	577.13385<br>631.05688	$[C_{30}H_{25}O_{12}]^-$<br>$[C_{27}H_{19}O_{18}]^-$	0.72<br>0.19	451, 425, 407, 289, 245, 161, 125<br>445, 301, 275, 167	280	Procyanidin B4
7	16.19	577.13477	$[C_{30}H_{25}O_{12}]^-$	1.1	451, 425, 407, 289, 245, 161, 125	279	Procyanidin B2
8	16.80	289.07219	$[C_{15}H_{13}O_6]^-$	1.53	245, 205, 179, 125	279	Epicatechin
9	16.97	647.05215	$[C_{27}H_{19}O_{19}]^-$	0.65	575, 433, 405, 235, 169	280	Ellagic acid xyloside derivative
10	17.03	633.07137	$[C_{27}H_{21}O_{18}]^-$	-0.87	463, 301, 275, 169	274	Galloyl-HHDP[c]-glucose
11	17.30	517.55884<br>1036.1166<br>387.16497	n.d. (2^-)<br>$[C_{18}H_{27}O_9]^-$	-<br>0.01	354, 301, 169<br>207	276	Ellagitannin<br>Phenolic glycoside
12	17.75	461.16516	$[C_{20}H_{29}O_{12}]^-$	-0.19	415, 269, 161, 101	279	Phenylpropanoid
13	18.41	516.54807<br>1034.0934	n.d. (2^-)<br>n.d.	-	301, 275, 169, 123<br>301	280	Ellagitannin
Fractions 7-11 (Fig. 7B)							

7	16.19	577.13477	$[C_{30}H_{25}O_{12}]^-$	1.1	407, 289, 245, 161, 125	278	Procyanidin B2
8	16.80	289.07219	$[C_{15}H_{13}O_6]^-$	1.53	245, 205, 179, 125	279	Epicatechin
10	17.03	633.07137	$[C_{27}H_{21}O_{18}]^-$	-0.87	463, 301, 275, 169	277	Galloyl-HHDP[c]-glucose
11	17.30	517.55884	n.d.	-	354, 301, 169	277	Ellagitannin
12	17.75	461.16516	$[C_{20}H_{29}O_{12}]$-	-0.19	415, 269, 161, 101	277	Phenylpropanoid
14	19.24	468.04458<br>937.08788	$[C_{41}H_{28}O_{26}]^{2-}$<br>$[C_{41}H_{29}O_{26}]^-$	-0.65	419, 275, 169, 125<br>Not available	278	Trigalloyl-HHDP[c]-glucose
15	19.80	475.18207	$[C_{21}H_{31}O_{12}]^-$	-0.03	301, 275, 247, 169, 125	279	Unknown ellagic acid derivative
16	20.36	393.04651<br>787.09924	$[C_{34}H_{26}O_{22}]^{2-}$<br>$[C_{34}H_{27}O_{22}]^-$	0.18<br>-0.71	169, 125<br>618, 465, 295, 169, 125	279	Tetragalloylglucose
17	21.55	469.05231<br>939.10553	$[C_{41}H_{30}O_{26}]^{2-}$<br>$[C_{41}H_{31}O_{26}]^-$	0.50<br>-5.38	169<br>169	280	Pentagalloylglucose

Notes:

[a] Numbers refer to peaks in Fig. 7 (ranked according to elution time). Numbered peaks occur in at least two fractions. Some compounds occur in both Fig. 7A and 7B and have the same numbering in both groups.

[b] The difference between the calculated molecular weight of the ion/compound and the observed molecular weight.

[c] Hexahydroxydiphenoyl.
